# Ciaran Mulholland: the Psychiatrist's Manifesto

**DOI:** 10.1192/bjb.2020.52

**Published:** 2020-10

**Authors:** Claire Mckenna

**Figure:**
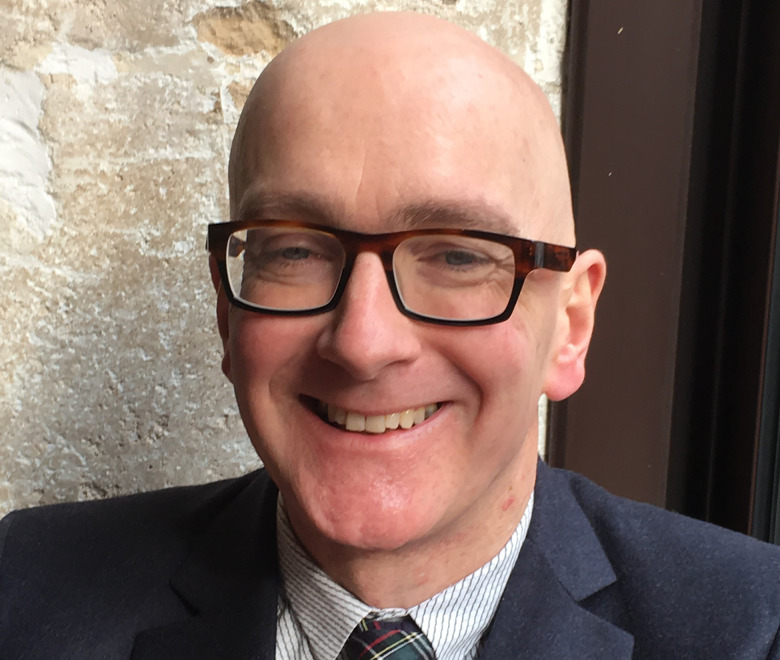

London-based neuropsychologist Vaughan Bell recently mused on Twitter: ‘I'm always slightly mystified why the situation in Northern Ireland gets ignored by the wider UK mental health community. Thirty plus years of armed conflict, highest prevalence of mental health problems in the UK, shockingly low level of investment in services’ (@vaughanbell, 2 Feb 2020; Tweet reproduced here with permission). I confess, I myself have wondered.

Inequality is indeed writ large in the mental health of Northern Ireland's populace. The statistics are alarming: deaths by suicide have doubled since the ceasefire heralded by the Good Friday Agreement in 1998 and are now the highest in the UK; there is a 25% higher prevalence of mental health problems in Northern Ireland compared with England; and Northern Irish people are prescribed more psychotropic medication than any other European population.

Professor Ciaran Mulholland took time out to talk to me during a 2019 conference on ‘Psychiatry and Conflict’ in Belfast, where he was speaking in his role as clinical director of Northern Ireland's unique Regional Trauma Network. Set up in 2017 as a fitting legacy for the 3700 killed and 55 000 injured in the conflict, it aims to provide a world-leading mental health service, across a network of voluntary and statutory sectors, for victims of all forms of trauma in Northern Ireland.

## A legacy of conflict and deprivation

Mulholland's first posting as a consultant adult psychiatrist was in 1998, the year the worst of the violence stopped. His base was the socially deprived area of Newtownabbey on the Northern outskirts of Belfast:

‘There was a lot of paramilitary activity in the area. There were also parts of the catchment area where many ex-soldiers, ex-policemen and ex-security forces were living, who clearly had their own experience […] A lot of what I dealt with was trauma related, where the individuals in some way have been impacted by the Troubles.’

Newtownabbey is a microcosm of Northern Ireland's more deprived communities. Many moved there to get away from the violence in Belfast in the 1970s, some involuntarily:

‘That means the local community is quite atomised and not as cohesive as you might get in some parts of Northern Ireland, which is one of the reasons Newtownabbey has quite high rates of psychiatric problems.’

Northern Ireland has higher rates of mental illness than the rest of the UK across all types of mental health conditions. Why have so many people not seen the dividends of peace reflected in their mental health? Mulholland says the causes are complex: socioeconomic deprivation is strongly associated with mental illness; there is a long legacy of underfunding of mental health services (half the proportion of health funding compared to England); there is a tribal political structure, leading to impasses in policy decision-making; and the psychological ripples of the Northern Ireland civil war continue to reverberate 23 years after it ended.

And then there are those stark suicide statistics: 143 people died by suicide in Northern Ireland in 1996. When last recorded in 2015, the figure had more than doubled to 318. It's tempting to attribute the lower rates in wartime to a protective ‘Blitz-spirit’, which bonds people together through shared adversity. Mulholland thinks this could be a factor in more deprived communities, but we have to be careful not to over-interpret.

He points out that during the years of the Troubles, paradoxically, policing by paramilitaries kept drug misuse in Northern Ireland lower than anywhere else on these islands – which is now not the case. There is also some evidence that, for people in their 50s or 60s, who lived through the worst years of violence, their untreated trauma was a factor in their suicide decades later. Most people who die by suicide in Northern Ireland don't have contact with mental health services, so tackling it can't be addressed by mental health services alone.

## PPE: politics, psychiatry and economics

We're talking in the Harland Bar, named after the local shipyard, in the shadow of the four hulking steel prows of Belfast's Titanic building. It seems an apt place to meet, for a man whose political life has been bound up with the struggles of the blue-collar worker:

‘Well, I mean, I am a socialist, you know. I'm on the left and my version of socialism is avowedly anti-sectarian. So, I've not needed to be in either camp. I've a very clear, distinctive view about there being a third tradition in Northern Ireland, which is kind of around labour and socialist politics.’

Northern Irish psychiatry is a small community, where reputations travel by word of mouth. Mulholland seems to plough his own furrow, more or less. Genial and unassuming in manner, the only signifiers of non-conformity are the Dr Martens boots he wears with his suit. There is no evidence of the reputed (possibly apocryphal) Trotskyite student streak.

Mulholland says that doctors in Northern Ireland have always had strong links with the socialist tradition. The nascent socialist and labour movement was stymied by the eruption of the civil war in 1968–1970. Sectarianism has dominated the political discourse ever since. He describes the influence of these socialist roots on his professional life:

‘It was just central to my work from the day I began working as a doctor, even before I trained in psychiatry. Perhaps that was of assistance to me that I wasn't as focused on the biology as a causative factor and as a sole explanatory factor, as maybe some other doctors were.’

In the Northern Ireland context, he thinks socialism has a unique ability to straddle the sectarian divide:

‘I think socialism properly speaking is neither Unionist nor Nationalist and seeks to unite people […] And it's around issues like health and education. That's how you unite people in terms of what they have in common as opposed to dividing them.’

Although for some people socialism is conflated with the ‘Red Peril’, Stalinism, et cetera, Mulholland is at pains to stress that fundamentally it's about creating a more equal society: ‘That's [Stalinism], not my version of socialism. It has to be democratic or it's not socialism’.

Mulholland sees the current resurgence of socialism (among young people in particular) as a renunciation of individualism. He detects a growing public awareness that collective purpose is needed to reduce social inequality and to improve our mental health:

‘I think it's the impact of the global crisis of 2007, 2008, 2009 […] that led to people who hadn't previously questioned the system, how it operated, asking profound questions. And I think that's now magnified by other developments, for example the question of the future of the planet, the environment […] if you study that particular threat carefully, and you think about what the alternatives are, it does suggest transformational change is required.’

## What is at psychiatry's cutting edge?

No, it's not the sexy neuroscience. ‘I suppose I've always been interested in prevention and early intervention’, Mulholland explains, ‘in the sense that if psychiatry has a cutting edge it's about: How early can we intervene?’

If circumstances hadn't changed suddenly in 2012 he might have stayed in his first consultant job in Newtownabbey. He collapsed at work, was rushed to theatre with a haemoglobin of 3 and was diagnosed with a rare form of stomach cancer. There was no Damascene moment, but after 9 months off, with time to take stock, he came back to find a small pot of money available to do some innovative work.

One of the earliest advocates in Northern Ireland for early intervention in psychosis services, he now works as a consultant psychiatrist with STEP (Service, Treatment, Education and Prevention), Northern Ireland's only early intervention service for the ‘at-risk mental state’, which he helped set up in the Northern Trust area. He is also one of the clinicians who has done most to fertilise the ground for mental health research in Northern Ireland. He co-leads the mental health special interest group in the Northern Ireland Clinical Research Network.

His research interests primarily focus on first-episode psychosis, prevention of transition to psychosis and the impact of trauma on mental health. Northern Ireland has higher rates of psychosis than most places in the UK (excepting parts of London with a high African–Caribbean population).

There is a burgeoning evidence base that early intervention can improve outcomes for young people with psychotic illness and even prevent transition from at-risk mental states in some cases. Though as Mulholland says, it can be quite difficult to prove a negative.

## The Belfast fixator

Northern Irish medicine has some grim claims to fame because of medics' unique war-zone experience. The ‘Belfast fixator’, for example, was designed here by an orthopaedic surgeon to heal injuries from beatings and explosions.

Four out of ten adults in Northern Ireland have been directly or indirectly affected by Troubles-related trauma, Mulholland says. According to data collected between 2004 and 2008, 8.8% of the population met criteria for post-traumatic stress disorder (PTSD) at some point in their lifetimes (higher even than in other, more intense conflict zones such as Lebanon and Israel).^1^ The methodology of that paper has been pored over since, but he backs up its findings:

‘It is clear and demonstrable that other places have higher rates of trauma with lower rates of PTSD. Some other factors must be at play […] one of the areas which we are compared to, for example, is Israel […] but there is evidence that Israeli society is particularly cohesive. That's a protective factor and perhaps our society is not quite so cohesive and that's how rates [of PTSD] have crept up.’

How then does a trauma history affect the trajectory of severe mental illness such as bipolar disorder and psychosis? When Mulholland was training, the dominant paradigm was a biomedical one, but he says,

‘I was always interested in the idea that actually psychological trauma was more central to the aetiology and the course of schizophrenia than was allowed for. And it was around that time in the 1990s […] that evidence began to emerge from groups all over the world, that psychological trauma can actually cause psychosis.’

Although this is more widely accepted now, he allows that there is much work to do to develop causal models of psychotic illness.

The Northern Ireland Regional Trauma Network, of which he is clinical director, was set up with a research focus from day one. Randomised controlled trials looking at epigenetic factors in the intergenerational transmission of trauma and psychological treatments of trauma are in the offing.

Mulholland discusses the emerging evidence that psychological trauma causes brain damage, particularly to the hippocampus and amygdala. He says there is evidence that damage can be repaired with medication and psychological therapy: ‘So a psychological insult causes brain damage and psychological therapy causes brain repair. I think that's very exciting and it opened up an entirely new way of looking at the brain’.

How does he respond to critics who, on the basis of the association between trauma and mental illness, question the validity of a biological contribution to psychiatric disorders? He suggests that we avoid ideological entrenchment:

‘I would have an issue with the individuals who practise biological determinism […] but I think equally now we have strident voices where it's psychological determinism, where it's your psychology that determines everything, and that's clearly not always the case.’

## Media and mental health

Mulholland has long had an interest in the portrayal of mental health in film and media. He helped organise a symposium on ‘Film and the politics of mental health’ in 2016, with a focus on two countercultural figures, Franco Basaglia and R.D. Laing. I wonder whether I've caught a glimpse of the radical psychiatrist within, but he remains elusive.

Mental health anti-stigma messages sometimes seem at saturation point in the media, but it's not the ‘worried well’ Mulholland wants to target:

‘I think it's one role of psychiatrists to ensure that there's greater understanding that there is such a thing as a mental illness. I think that actually is important. You could say it's a concept or a construct and it is in a sense, of course, but I think it's important for us to make the case that sometimes there is demonstrable pathology, which means that a person's way of being is different, which meets all the criteria for illness and that ought to be addressed.’

In a socially conservative society like Northern Ireland he thinks this is especially crucial. The BBC's Countryfile programme shone a spotlight on mental health problems among gay farmers last year. Mulholland was involved with the episode, which interviewed a Northern Irish farmer who had suicidal feelings as a result of repressing his sexual identity.

Since then he has been working with the BBC on a Horizon programme (screened in spring 2020), about prominent 1990s comedian Tony Slattery. In particular, he wants to highlight the genesis of mental illness through trauma:

‘He [Slattery] received a diagnosis of bipolar affective disorder after his parents died. He went into the media and began to talk about a traumatic experience at the age of 8 and once he began to talk about it, he wanted to explore it further. So we worked with the producers of Horizon and made a programme all around this, about his life trajectory, his life course, but going beyond that to examine bipolar disorder as a concept – what the biological underpinnings of bipolar disorder might be and the role of trauma in bipolar disorder.’

## Not another brick in the wall

Early-life adversity remains the fulcrum of Mulholland's interests. Northern Ireland has higher levels of multiple deprivation (defined as an inability to access three or more basic necessities) than the rest of the UK. The most deprived areas are also those most affected by the Troubles.^2^ He references the zeitgeisty book *The Spirit Level*,^3^ which made a compelling case that socio-economic inequality, rather than poverty per se, drives poor mental health:

‘So in the United States and the United Kingdom, where neoliberal ideas were most to the fore, appear to have higher rates of mental problems compared to more cohesive societies like the Scandinavian countries where there are lower rates of social division.’

We discuss economist Lord Layard's seminal 2006 report, which led to the Improving Access to Psychological Therapies (IAPT) programme in England. Among the criticisms of Layard was his framing of anxiety and depression as discrete illnesses that, when individually treated could return people to economic productivity, rather than formulating these problems as people's responses to the difficult socioeconomic circumstances in which they find themselves.

Mulholland demurs:

‘I think in the main it's [IAPT] been an excellent initiative that has brought psychological therapies to large numbers of individuals who've benefited and who wouldn't have received such treatment. I think Northern Ireland would benefit from a similar initiative.’

In the manner of Sophie's choice, I ask him to pick one thing that would have the biggest impact on mental well-being in Northern Ireland. A glimpse of the radical psychiatrist emerges at last:

‘Our society is dysfunctional. It's a function of the level of division in society that we are literally divided by walls, physical walls [the incongruously named ‘peace walls’ that separate Catholic and Protestant communities], but we're also divided by metaphorical walls in various ways. There's clear evidence that those who live close to the physical walls, the ‘peace lines’ that separate our communities, have higher rates of mental health problems.

‘So, in Northern Ireland […] in my working lifetime, the number of consultant psychiatrists has doubled. Where's the demonstrable impact on the rates of mental health problems? Can we prove that we are effective? I think there's a real challenge there. Whereas we can demonstrate that if you make real changes to societal structures, that *will* have an impact on rates of mental health problems.’

Long live the (democratic, peaceful) revolution.
